# Dispensary of Jefferson Medical College

**Published:** 1844-05-18

**Authors:** Edward R. Squibb


### CLINICAL LECTURES AND REPORTS.

### DISPENSARY OF JEFFERSON MEDICAL COLLEGE.

### PROFESSOR BACHE'S CLINIC (MEDICAL.)

[Reported by Edward R. Squibb.]

*Wednesday, April* 24, 1844.

Case I.—Ann McG., æt. 28. Complains of pain and swelling of the hand and arm; commenced by numbness in the fingers, extending upwards, and becoming painful; feels most relief when holding it down as low as possible; has been affected for some time; has no use whatever of either the hand or arm; bowels regular; general health and appetite good.

Dr. Bache supposed this to be a case of partial rheumatism, for which she was ordered to be bled 12 oz. and take of the wine of colchicum root 10 drops every two hours; the hand and arm to be rubbed with volatile liniment.

Case II.—Elizabeth R. colored, æt. 52. Has pain in the side and breast; shortness of breath; cough, which is increased by the horizontal position, and palpitation of the heart on making exertion; tongue pale; appetite middling; rests badly; looks quite fleshy, but complains of weakness; bowels irregular; was ill some time since, of an affection of the chest, for which she got bled.

This patient appeared to be suffering from an effusion, the result, probably, of an ill-treated pectoral affection; directed to take a purge, viz:—

Potassæ Bitart. ʒj.

Pulv. Jalap. grs. x.

Every day, and as an anodyne 10 grs. of powdered ipecac. and opium every night.

Case III.—Miss Jane G., æt. 42. Always enjoyed good health until within the last three weeks, during which time she has suffered from a bad headache, itching of the lower extremities, and loss of appetite, and loss of power in her arms, not being able to use them as usual; rests well; is somewhat feverish; bowels constipated; pulse frequent.

Ordered to have 12 oz. of blood taken, and as a purgative to take three compound cathartic pills at bed time.

Case IV.—John A., æt. 22, weaver. Looks pale, and somewhat emaciated; was affected with constipation some four weeks since, when he took salts and castor oil without being much relieved; has a hard cough, which is worse through the day, the expectoration being slight; loss of appetite, bad rest, frequent pulse, night sweats, and a bad taste in the mouth; tongue appears nearly natural; has felt chilly for four days past; been working in a damp cellar.

Advised to abandon weaving altogether, and when he gets better, adopt some other occupation.

Ordered three compound cathartic pills as a purgative to be taken immediately, and to relieve the cough, and procure rest, the following mixture:—

℞. Pulv. Ext. Glycyr.

Pulv. Acaciæ *aa* ʒij.

Vin. Antim. f.ʒij.

Tinct. Opii gtt. xl.

Aquæ f.℥iv.

M. A tablespoonful to be taken occasionally.

Case V.—Thomas A., æt. 34. First came under treatment on Wednesday, April 17th, at which time the prominent symptoms were pain in the side and breast; a harassing cough, with bloody expectoration; very frequent pulse, red tongue, and fever and sweating, with much emaciation. The treatment then ordered was, as an alterative, three grs. of iod. potassium three times a day in solution; a plaster of burgundy pitch with cantharides to be applied over the seat of pain; and to allay irritation and procure rest, a teaspoonful of the solution of sulphate of morphia at bed time. To-day the patient feels somewhat better; expectoration more free; rests better; pulse 152.

Directed to continue the treatment.

Case VI.—James C., æt. 26. Has been under treatment for an affection of the spine in the lumbar region, the prominent symptoms of which were constant pain in the location indicated, attended with slight fever, loss of appetite, and constipation.

This day two weeks, was ordered to be cupped over the seat of pain. This measure affording no relief, he was ordered at the end of a week, to apply the following ointment to the spine, viz:—

℞. Veratriæ grs. v.
Adipis ʒij.
M. And to take a purge of three compound cathartic pills.

To-day complains of a slight cough; of resting badly, for the most part in the sitting posture, as the only one in which he is easy. Papillæ of the tongue inflamed.

Ordered to change the treatment to twenty drops of the wine of colchicum root to be taken every two hours, increasing or diminishing the dose as the patient may bear it.

CASE VII.—Mary T., æt. 47. Has been under treatment for habitual vomiting and constipation, for which she was ordered to take a teaspoonful of white mustard seed three times a day, with one compound cathartic pill every night.

As she has experienced little benefit, was ordered to abandon the mustard seed, to increase the cathartic to two pills every night, and to take five grs. of the subnitrate of bismuth, three times a day.

CASE VIII.—Mrs. Eliza S., æt. 33. Complains of a bad cough, which is most troublesome at night; pain in the side and back, and shortness of breath; rests badly; bowels somewhat constipated; pulse natural; tongue whitish.

Ordered to take the liquorice mixture prescribed for case IV, and apply a plaster of Burgundy pitch and cantharides over the seat of pain.

